# Toward a Selective Analysis of Heavy Metal Salts in Aqueous Media with a Fluorescent Probe Array

**DOI:** 10.3390/s22041465

**Published:** 2022-02-14

**Authors:** Andrey G. Melnikov, Denis A. Bykov, Alexey S. Varezhnikov, Victor V. Sysoev, Gennady V. Melnikov

**Affiliations:** Department of Physics, Yuri Gagarin State Technical University of Saratov, 410054 Saratov, Russia; robokop2468.bykov@yandex.ru (D.A.B.); alexspb88@mail.ru (A.S.V.); melnikov_gv@sstu.ru (G.V.M.)

**Keywords:** fluorescence sensor, heavy metal, multisensor array, selectivity

## Abstract

Detection of heavy meals in aqueous media challenges worldwide research in developing particularly fast and affordable methods. Fluorescent sensors look to be an appropriate instrument for such a task, as recently they have been found to have made large progress in the detection of chemical analytes, primarily in the environment, along with biological fluids, which still suffer from not enough selectivity. In this work, we propose a new fluorescent method to selectively recognize heavy metals in an aqueous solution via employing an array of several fluorescent probes: acridine yellow, eosin, and methylene blue, which were taken as examples, being sensitive to a microsurrounding of the probe molecules. The exemplary sensor array generated six channels of spectral information through the use of various combinations of excitation and detection wavelengths. Following the known multisensor approach, we applied a linear discriminant analysis to selectively distinguish the vector signals from the sensor array from salts of heavy metals—Cu, Pb, Zn, Cd, and Cz—at the concentration ranges of 2.41 × 10^−6^–1.07 × 10^−5^ M, 2.8 × 10^−5^–5.87 × 10^−4^ M, 1.46 × 10^−6^–6.46 × 10^−6^ M, 1.17 × 10^−8^–5.2 × 10^−8^ M, and 2.11 × 10^−6^–9.33 × 10^−6^ M, respectively. The suggested approach was found to be promising due to it employing only one cuvette containing the test solution, simplifying a sample preparation when compared to preparing a variety of solutions in tests with single fluorescence probes.

## 1. Introduction

At present, human activity leads to an increase in environmental pollution by various ecotoxicants including heavy metals. In this regard, the development and implementation of new methods that could allow one to register their presence are rather urgent [1]. There are three major types of environmental pollution, namely, in the atmosphere [2,3], in water [4], and in soil [5,6], of which liquid media is possibly the most challenging. Indeed, while some heavy metals have important biological functions in plants, animals, and humans [7], their chemical structure and redox properties lead sometimes to the fact that they can avoid mechanisms of their excretion from the body, such as homeostasis transport by proteins and binding to the necessary components of the cell [8]. These metals bind to regions of the protein, displacing the parent metals in their natural binding sites that cause malfunctioning of cells and has a toxic effect. For instance, previous studies have shown that an oxidative degradation of biological macromolecules appears primarily due to the binding of heavy metals to DNA and nuclear proteins [9]. Therefore, heavy metals are considered to be large toxicants that may damage multiple human organs even at low exposure levels. They are also classified as carcinogens to humans, known or probable, by the U.S. EPA and the International Agency for Research on Cancer [10]. Altogether, an early detection of heavy metal ions is considered an important task in order to protect the environment from pollution [11].

The conventional analytical methods capable of detecting metal ions primarily employ atomic absorption or emission spectroscopy (AAS/AES) [12], inductively coupled plasma mass spectrometry (ICP-MS) [13], anodic voltammetry [14], and capillary electrophoresis [15], which normally require expensive equipment and/or rather complex sample preparation, making it difficult to detect the ions in situ and under a real timescale. For example, atomic absorption spectroscopy (AAS) is widely utilized for detecting traces of heavy metal ions in all types of liquid samples such as food [16] and biological samples [17,18]. Ordinarily, it measures an individual chemical element in the course of a single measurement [19]. Unlike AAS, inductively coupled plasma (ICP) spectrometry is a multi-element analysis technique [20,21]. The major challenges while applying ICP techniques are the difficulties in separating the target isotopes due to a noise in the detectors to be accompanied by a relatively long time for the analysis. Further, laser spark emission spectrometry (LSES or LIBS) yields a rapid analysis of heavy metals in water as well as continuous monitoring of analytes via analyzing their spectral characteristics [22]. It makes it possible to simultaneously perform multi-element analysis with a high sensitivity of up to 2 ppm [23,24]. However, the formation of dissolved gases and bubbles by laser pulses can often lead to defocusing of the laser beam, limiting LIBS application thus far [11]. The most sensitive technique is based on neutron activation analysis (NAA), which employs the material’s bombardment by neutrons in order to induce radioactive isotopes at a short half-life [25,26]. This method provides options to simultaneously identify many chemical elements without a chemical separation with a high sensitivity, up to 10ths of a nanogram per liter [27]. However, still, the NAA technique is difficult to employ in practice because the analysis requires radioactive substances that need special care. Finally, the potentiometric methods have attracted a considerable interest recently as an easier alternative with many possibilities for machine processing [28]. In particular, the voltammetric methods are widely employed in labs to register heavy metals [29,30]. However, in addition to high sensitivity, the potentiometric sensor arrays require special electrodes to be constantly monitored for their condition, which again limits their application.

Herein, we consider the technique that is matured from luminescent analysis to deliver information about the presence of heavy metals in a non-destructive manner, being extremely important for the analysis of biological objects [31,32]. This method employs a decreasing in the fluorescence intensity with the presence of heavy metals in the solution. Such a quenching is thought to be a result of intersystem crossing to an excited triplet state promoted by spin–orbit coupling of the excited (singlet) fluorophore and the halide upon contact [33], which is very sensitive to heavy metals contained in a solution [34]. In order for the selectivity of quantum yield of fluorescence to be advanced [35], there are two approaches, namely, (i) the molecular design of selective fluorescent probes [36,37,38,39] and (ii) applying new processing methods [40], which ensure the selective characteristics of fluorescence to a particular metal [41]; in particular, there is a high interest in designing multiband sensor array emission in a wide range of wavelengths [42]. The second direction involves using pattern recognition algorithms to process the vector signals generated by arrays of fluorescence sensors [43,44]. With such an option, it is possible to determine not only the presence of heavy metal ions but also to selectively recognize their kind. This method follows a general concept of the so-called “electronic tongue” [45,46], wherein fluorescent probes or quantum dots are selected so that the array includes probes that are sensitive to various metals. As a signal to process, the fluorescence intensities of the array are extracted to be further treated as a vector by, for instance, linear discriminant analysis (LDA) algorithm, one of the most powerful techniques for pattern recognition [47,48]. Similar LDA processing has been used when considering colorimetric methods to distinguish heavy metal ions in solutions [49]. Still, it is worth noting that utilization of a variety of solutions of the sample under study causes difficulties in the sample preparation and imposes advanced requirements to the initial volume of the sample. Furthermore, there is an effort to develop multisensors based on bacteria [50] in order to analyze the content of heavy metal ions in aqueous media, wherein researchers have used the influence of heavy metals on the growth of several selected cultures of bacteria. However, the noted measurements take a rather long time of around two days. Another example has been reported in [51], where the authors employed a multi-sensor composed of a number of synthesized fluorescence probes to selectively detect many compounds. Therefore, there is still a call for developing a method for a fast but selective recognition of heavy metal ions combined with a simple preparation of a sample.

**Figure 1:**
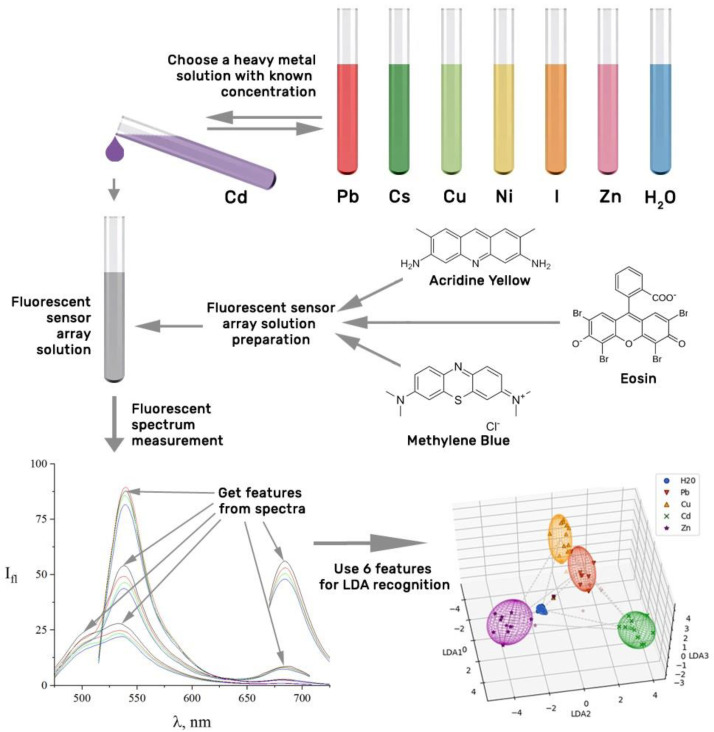
General scheme of the methodological approach.

In this work, we propose a sensor array based on three fluorescent probes to meet the noted call accounting for our preliminary findings [52], following a general scheme of the approach shown in Figure 1.

As we show, the proposed multisensor array, a mixture of luminescent probes, yielded a multiple fluorescence response to heavy metal ions in a liquid in accordance with their concentration at the microenvironment of the dyes. The kind of the ions is selectively distinguished by LDA processing of the vector signals taken from the array after the calibration of the array upon to the ion impact.

## 2. Materials and Methods

**Figure 2:**
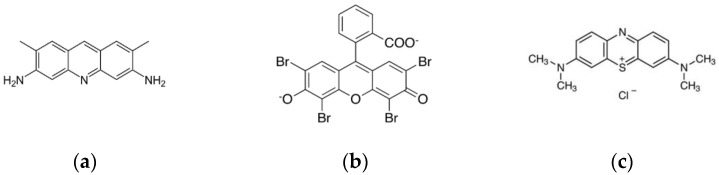
Structural formulas of dyes employed in the work to create the fluorescent probe array: (**a**) acridine yellow (ϕ = 0.47, λ_ex_ = 445 nm, λ_em_ = 505 nm), (**b**) eosin (ϕ = 0.67, λ_ex_ = 500 nm, λ_em_ = 545 nm), and (**c**) methylene blue (ϕ = 0.52, λ_ex_ = 660 nm, λ_em_ = 680).

To show the feasibility of the method, we considered three fluorescent dye probes, namely, eosin yellow, acridine yellow, and methylene blue (Sigma-Aldrich Pty Ltd., Darmstadt, Germany). These dyes have long been used for fluorescence studies due to their high sensitivity to changes on their microenvironment. For instance, eosin Y, bound to ligands, has been frequently used to register Co, Ag, Zn, Cd, and Pb ions via making triple complexes with these metals and ligands [53]. Moreover, eosin is widely employed in electrochemistry to create electrodes sensitive to Cd [54]. The acridine dyes and their derivatives are widely used as fluorescent and electrochemical sensors [55,56,57], as solar photocatalysts for enhancing biodegradability of toxic compounds [58], and for development of new fluorescent pH sensors [59]. Methylene blue is widely used in medicine [60] and as a near-infrared fluorescent probe [61,62]; its polarization is used as a quantitative marker of cancer at the cellular level [63]. The Figure 2 depicts the structural formulas of these compounds.

These substances are common luminescent probes with a high quantum yield of fluorescence, being ϕ = 0.47 for acridine yellow [64], ϕ = 0.67 for eosin [65], and ϕ = 0.52 for methylene blue [66]. Moreover, these fluorophores yield various wavelengths of their fluorescence, which is a prerequisite for the successful and simple separation of signals to form a sensor array. When considering the concentration values of dyes, we accounted for following conditions: (i) exclusion of the influence of the internal filter, and (ii) the fluorescence maxima of the dyes upon excitation at a wavelength of 260 nm, which should appear approximately at the same level. The dye solutions were prepared by diluting the pristine dry matter in a distilled water with further final adjusting of these initial solutions with water. The photochemical activity of the dyes was reduced to a minimum by maintaining a constant temperature and pH. We controlled the process by measuring the absorption spectra and extracting the concentrations according to the Bouguer–Lambert–Beer law. Final concentrations of acridine yellow, eosin Y, and methylene blue were 1.13 × 10^−7^, 9.21 × 10^−9^, and 9.32 × 10^−7^, respectively. Final concentrations of Cu, Pb, Zn, Cd, and Cz were in the ranges of 2.41 × 10^−6^–1.07 × 10^−5^ M, 2.8 × 10^−5^–5.87 × 10^−4^ M, 1.46 × 10^−6^–6.46 × 10^−6^ M, 1.17 × 10^−8^–5.2 × 10^−8^ M, and 2.11 × 10^−6^–9.33 × 10^−6^ M, respectively.

In the course study, we took several basic solutions of heavy metal salts, copper (Cu(NO_3_)_2_), lead (Pb(NO_3_)_2_), zinc (Zn(NO_3_)_2_), cadmium (Cd(NO_3_)_2_), and cesium (CsCl). These solutions have been prepared by diluting a required amount of dry matter (salts) in distilled water. To study the response of the fluorescent probes in the array to the presence of heavy metal ions, we added minor volumes of solutions, 0.1–0.5 mL, containing these salts into the liquid containing the sensor array, of 3 mL volume, with further recording of the fluorescence spectra. These concentrations were chosen following a number of preliminary studies to be at the minimum level able to detect with the employed dyes. While performing the study, we tried to avoid photo-bleaching of the dyes by their proper conditioning: (i) the solutions were kept in a fridge in a light-isolated box; (ii) the time to prepare the probes and to measure the spectra of each sample was the same; (iii) the addition of salts of heavy metals to solutions of multi-probe array were prepared just before the experiment; and (iv) the time to record the spectra was chosen to exclude the spectra distortions.

The dye fluorescence spectra were measured with a high-resolution spectrometer (Perkin Elmer LS-55, PerkinElmer, Inc., Waltham, MA, USA). Excitation and emission slits were set to 10 nm, and excitation wavelengths were varied in the range of 260–660 nm as described below. At an excitation wavelength of 260 nm, we used an in-built emission filter at 350 nm.

For the processing of vector signals generated by a fluorescent probe array, we used the LDA algorithm taken in the standard Python libraries of the scikit-learn package [67].

## 3. Results and Discussion

The major issue of concern in this work relates to advancing a selectivity of detecting metal ions in aqueous media with multiple fluorescent probes. The fluorescence of each luminescent probe taken to combine the array is a separate source of information on changes in the dye microsurrounding. The addition of several probes together with the solution makes possible to obtain more data in a single measurement. To yield the number of features from the fluorescent probe array for the ion recognition, we performed a fluorescence excitation at different wavelengths in the course of spectral measurements.

**Figure 3:**
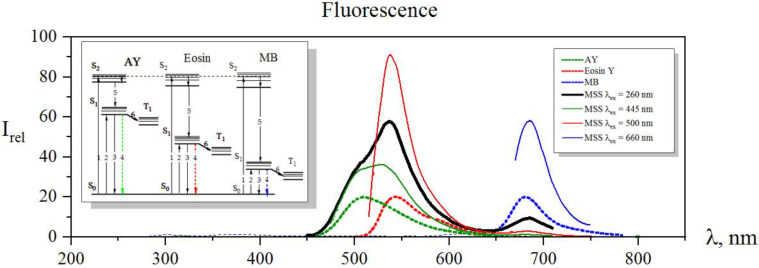
The fluorescence spectra of a fluorescent probe array under excitation at various wavelengths of 260 nm (black solid line), 445 nm (green line), 500 nm (red line), and 660 nm (blue line). The dotted lines mark fluorescence spectra of acridine yellow (green dotted), eosin (red dotted), and methylene blue (blue dotted), normalized to I_rel_ = 20. The insert is an energy diagram illustrating the photophysical processes taking place in the system.

Figure 3 shows the fluorescence spectra of individual dye solutions and the fluorescence spectrum of their resulting mixture, a multiple probe array, upon excitations at 260 nm, 445 nm, 500 nm, and 660 nm wavelengths.

These excitation wavelengths were chosen as follows: At a wavelength of 260 nm, all dyes of the array had an absorption peak related to S_0_-S_2_ transfer. Upon excitation, the electrons went to the S_2_ excited level, as drawn in Figure 3, to be a process 1 at the energy diagram. Then, they dropped down to the S_1_ state as a result of internal conversion (process 5) and further yielded a fluorescence (process 4), which was recorded by the spectrophotometer. In this case, fluorescence bands of all the three dyes were observed in the spectrum, and maxima of fluorescence intensities of the probe array were recorded at wavelengths of 505 nm, 540 nm, and 685 nm. These intensity values were employed as three primary features for the analyte recognition. When the fluorescent probe array was excited at wavelengths of 445 nm, 500 nm, and 660 nm, the dyes went into the S1 excited state (process 2) with subsequent appearing of fluorescence (process 4) again. These intensities of the maxima of all three probes were recorded with a spectrophotometer as secondary three features for an analyte recognition. Altogether, we had six features that could be employed for a sensitive response to changes in the microsurrounding of the probes. From a fundamental viewpoint, when ions of heavy metals in a liquid meet a dye molecule, the spin of the valence electron of the dye flips and it goes to a triplet state (process 6). As a result, the population of the S_1_ state and the number of fluorescence transitions (4) reduced, and we observed a fluorescence quenching [33]. Thus, the influence of heavy metal ions on the fluorescent probes manifested itself upon excitation of the system in both S_1_ and S_2_. However, the quenching efficiency strongly depends on the nuclear charge of a metal atom: as a first approximation, the larger charge results in greater efficiency, making a fundamental background for the ion selectivity by analyzing a vector signal of the probe array.

**Figure 4:**
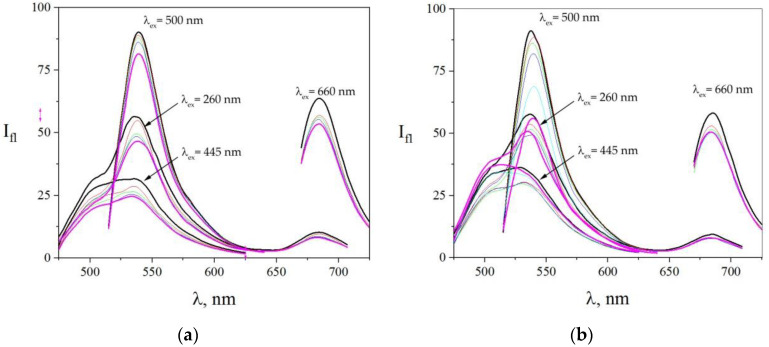
The spectra of the fluorescent probe array after adding heavy metal ions to a solution, subject of excitation at various wavelengths, 260–660 nm. (**a**) Case of [Cu^2+^], wherein the concentrations of the salt were C_Cu(NO3)2_ = 0 M (black line), C_Cu(NO3)2_ = 2.41 × 10^−6^ M (red line), C_Cu(NO3)2_ = 4.67 × 10^−6^ M (green line), C_Cu(NO3)2_ = 6.8 × 10^−6^ M (blue line), and C_Cu(NO3)2_ = 1.07 × 10^−5^ M (purple line). (**b**) Case of [Pb^2+^], wherein the concentrations of the salt are C_Pb(NO3)2_ = 0 M (black line), C_Pb(NO3)2_ = 2.8 × 10^−5^ M (red line), C_Pb(NO3)2_ = 7.9 × 10^−5^ M (green line), C_Pb(NO3)2_ = 1.24 × 10^−4^ M (blue line), C_Pb(NO3)2_ = 3.26 × 10^−4^ M (aquamarine line), and C_Pb(NO3)2_ = 5.87 × 10^−4^ M (purple one). The excitation wavelengths are shown in the figure.

Figure 4a displays spectra recorded under copper salt being added to the dye solution. As we see, a fluorescence quenching occurred in all the dyes of the array. The presented spectra showed that the quenching rate varied at different spectral fluorescence bands: for excitation on 260 nm, acridine yellow peak decreased from 32 a.u. to 22 a.u., that is, by 30%; eosin peak decreased from 56 a.u. to 46 a.u. (18%); and methylene blue peak fell from 10 a.u. to 8 a.u. (20%). It can be seen from the figure that acridine yellow and methylene blue fluorescence quenching went more rapidly than for eosin. For excitation at acridine yellow, the absorption peak at around 445 nm of its fluorescence maximum went down from 28 a.u. to 20 a.u., that is, by 28%. For excitation at eosin yellow, absorption peak at around 500 nm of its fluorescence was reduced from 90 a.u. to 81 a.u. (28%). When the excitation wavelength was set to 660 nm, which corresponded to methylene blue absorption maximum, we observed quenching of its fluorescence from 64 a.u. to 53 a.u. (17%). This shows the relevance of using multiple excitation wavelengths to increase the variability of the fluorescent probe array.

The similar processes are observed when the lead salt has been added to the solution of the fluorescent probe array. The corresponding spectra changes are drawn in Figure 4b. We note a reduction in the fluorescence of eosin (from 91 a.u. to 56 a.u., that is, 39%) and methylene blue (from 58 a.u. to 50 a.u., that is, 14%), while the fluorescence of acridine yellow is first quenched (from 33 a.u. to 27 a.u., that is, 18%) and then enhanced (from 27 a.u. to 37 a.u., that is, 37%), making a substantial difference to the Cu^+^ effect. Upon excitation at wavelengths of 450 nm, corresponding to its absorption maximum at around 260 nm, we observed an increase in its fluorescence upon addition of high lead concentrations from 3.26 × 10^−4^ M. This observed increase in fluorescence under high [Pb^2+^] concentrations cannot be explained by the effect of a heavy atom. However, there are some literature data showing that Pb can enhance fluorescence of probes under certain conditions [68,69,70].

**Figure 5:**
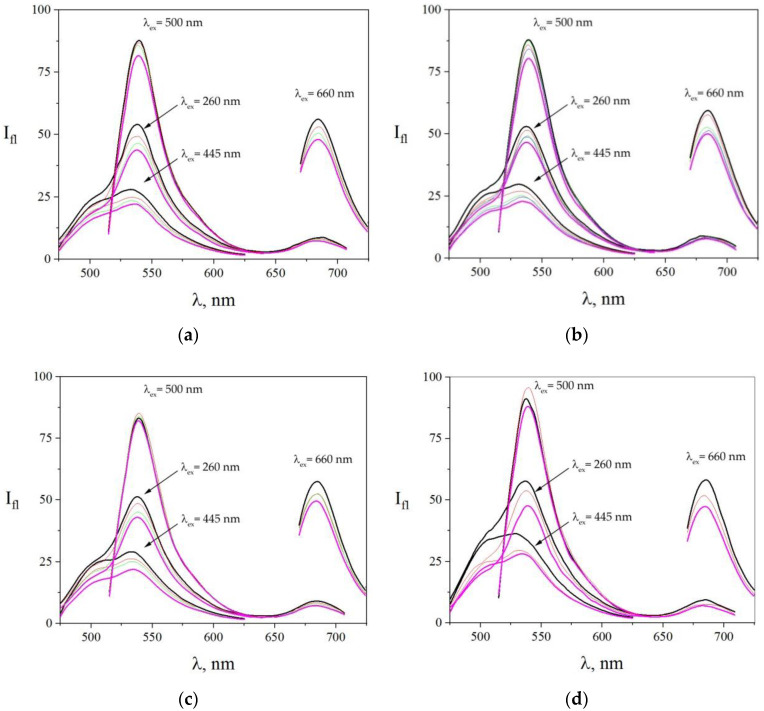
The spectra of the fluorescent probe array after adding heavy metal ions to a solution, subject to excitation at various wavelengths from 260 to 660 nm. (**a**) In the case of [Cd^2+^], the concentrations of the salt were C_Cd(NO3)2_ = 0 M (black line), C_Cd(NO3)2_ = 1.17 × 10^−8^ M (red line), C_Cd(NO3)2_ = 3.31 × 10^−8^ M (green line), and C_Cd(NO3)2_ = 5.2 × 10^−8^ M (purple line). (**b**) In the case of [Zn^2+^], the concentrations of the salt in the solutions were C_Zn(NO3)2_ = 0 M (black line), C_Zn(NO3)2_ = 1.46 × 10^−6^ M (red line), C_Zn(NO3)2_ = 2.83 × 10^−6^ M (green line), C_Zn(NO3)2_ = 4.11 × 10^−6^ M (blue line), and C_Zn(NO3)2_ = 6.46 × 10^−6^ M (purple line). (**c**) In the case of [Cs^+^], the concentrations of the salt were C_CsCl_ = 2.11 × 10^−6^ M (black line), C_CsCl_ = 2.11 × 10^−6^ M (red line), C_CsCl_ = 5.94 × 10^−6^ M (green line), and C_CsCl_ = 9.33 × 10^−6^ M (purple line). (**d**) In the case of various volumes of distilled water: 0 (black line), 1/30 of initial volume (red line), and 5/30 of initial volume (purple line). The excitation wavelengths are shown in the figure.

When adding Cd^2+^, Zn^2+^, and Cs^+^ ions to the solution of the dye array, the array response was also varied for each case. We summarize these findings in Figure 5. From adding Cd^2+^ ions, we found that fluorescence of all the three probes decreased: for acridine yellow from 22 a.u. to 16 a.u. (27%), for eosin from 88 a.u. to 81 a.u. (8%), and for methylene blue from 56 a.u. to 48 a.u. (14%). However, the rate of these quenchings was different. It can be seen from Figure 5a that acridine yellow and methylene blue fluorescence reduced more uniformly than with eosin. Contrastingly, the addition of Zn^2+^ ions (Figure 5b) suppressed the eosin fluorescence in another way. For comparison, as drawn in Figure 5c, the Cs^+^ ions essentially did not quench the eosin fluorescence when excitation was set as 500 nm, but we observed a noticeable quenching (16%) of eosin fluorescence when excitation was set to 260 nm. The acridine yellow and methylene blue peaks were quenched by 30% and 14%, respectively.

The distilled water was taken as a control sample for comparison to water not contaminated with heavy metal ions. The corresponding fluorescence spectra observed from the dye array are given in Figure 5d. As one can see, the spectra were still changed during the dilution but with different behavior when compared to metal salt solutions.

Overall, the results of spectral studies indicate that the nature of the reduction in the fluorescence intensity of the probed varies for different quenchers and upon dilution in pure water. Namely, the rates of quenching in dyes depended significantly on the type of heavy metals that yielded an opportunity to selectively identify them via collecting a total vector response of characteristic features observed for each dye in the array.

On the basis of the recorded spectra, we compiled tables of features corresponding to the response of the dye array under interaction with different metals. Each data point was normalized by the following formula:

(1) $$I=\frac{I_{Add}}{I_{MS}},$$

where *I_MS_* is a fluorescence intensity of the probe array without any addition, and *I_add_* is a fluorescence intensity of probe array upon the addition of test solutions under investigation.

**Figure 6:**
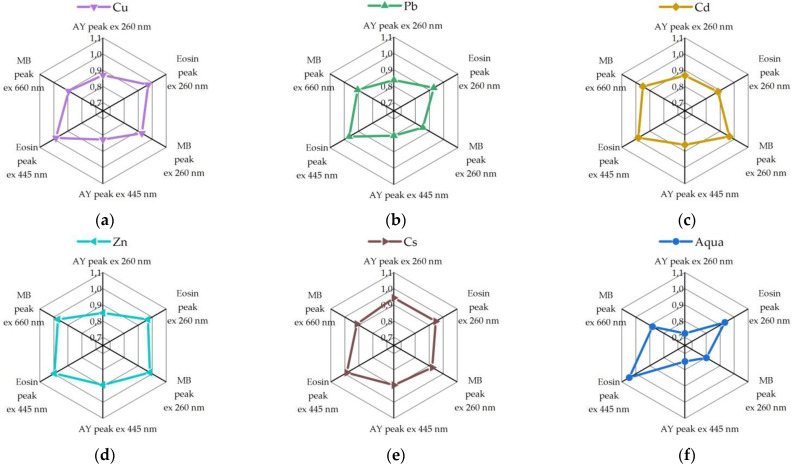
Normalized spectral responses (maxima of fluorescence intensities of three dyes upon excitation at 260 nm and at the maximum absorption of each dye) of the fluorescent probe array to various metal ions: (**a**) CuNO_3_ (C_Cu(NO3)2_ = 2.41 × 10^−6^ M), (**b**) PbNO_3_ (C_Pb(NO3)2_ = 2.8 × 10^−5^ M), (**c**) CdNO_3_ (C_Cd(NO3)2_ = 1.17 × 10^−8^ M), (**d**) ZnNO_3_ (C_Zn(NO3)2_ = 1.46 × 10^−6^ M), (**e**) CsCl (C_CsCl_ = 2.11 × 10^−6^ M), (**f**) H_2_O. The pristine fluorescence intensities of the dyes in array are normalized to 1.

These data points are compiled in Figure 6 to allow us easier comparison regarding their metal selective sensing capacity. The fluorescence intensities of the dyes were normalized to unity for further use as features of the test sample to combine a vector signal for pattern recognition processing via LDA algorithm. During the calibration of the algorithm, we used the experimentally obtained data sets, from which we obtained patterns corresponding to the following recognition classes: heavy metals of Cu, Pb, Cd, Zn, Cs, and pure water.

For each recognition class, 20 sets of initial data were used for training by introducing random errors into the initial data. According to the idea of the LDA algorithm, the projection is carried out in such a way as to ensure the maximum separation of classes regarding a scatter of data within a single class [71].

**Figure 7:**
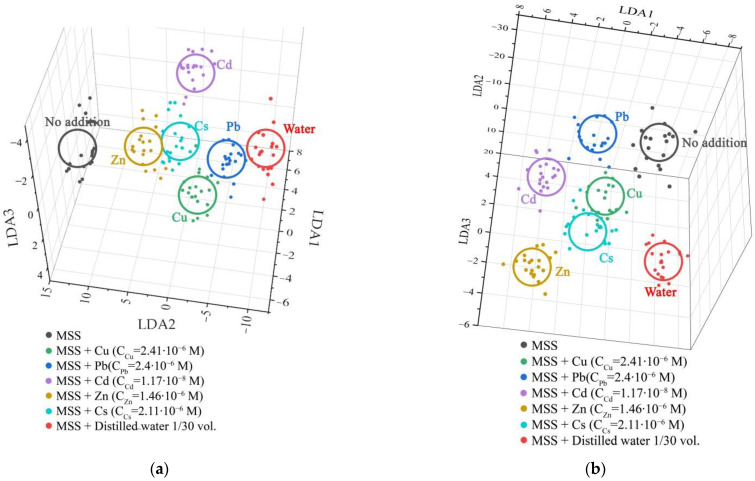
The 3D projection of the first three LDA components of 6D LDA coordinate system built to recognize vector signals of the fluorescent probe array versus seven test analytes added to 3 mL of solution containing the fluorescent probe array: (**a**) 0.1 mL volume of each test analyte; (**b**) 0.5 mL volume of each test analyte. The salt concentrations are drawn in the figure.

As a result of the learning process of the algorithm, the vector data are grouped into clusters corresponding to a particular type of analyte. By the distance between gravity centers of the clusters, one can judge the reliability of the classification. The LDA diagram that classifies the recorded data versus addition of 0.1 mL and 0.5 mL of each tested analyte solution to 3 mL solution containing the fluorescent probe array is shown in Figure 7. Here, we drew the 3D projection of the total 6D LDA coordinate system. It can be seen from the figure that the points on the diagram are grouped into clearly distinguishable clusters related to various analytes, which makes it possible to apply the methods of automatic signal recognition for their analysis. Thus, the LDA results show that the proposed system can be successfully used for selective recognition of heavy metal salts in water.

The experiments performed and the results of data processing allowed us to conclude that the fluorescence intensity of the luminescent dyes composing the fluorescent probe array was sensitive to the kind of various heavy metal ions to the solution. Due to the influence of various photophysical processes on the fluorescence process, changes in intensity were specific for various ions of heavy metals. Therefore, after the recognition algorithm was calibrated for the registration of predetermined substances, pattern recognition algorithms for the selective recognition of these substances in solution could be applied. Thus, this array can be employed for the selective determination of heavy metal salts in a liquid media.

The proposed approach is rather simple when compared to other ones, making it promising for practice. For instance, when using the above-noted methods [45,47,49], some difficulties are caused by the need to prepare a large number of solutions of the test sample with fluorescent probes. This requires a large volume of the investigated medium, which is not always possible to carry out, especially when determining heavy metals in a biological media. Here, we have a significant simplification of sample preparation, since only one solution of the sample under testing at a small volume, around 0.1 mL, was prepared, which is enough for a selective recognition.

## 4. Conclusions

Thus, in this work, a fluorescent probe array, consisting of a mixture of three luminescent dyes—eosin, methylene blue, and acridine yellow—was composed as an example of a low-cost ion-selective system. The fluorescence spectra were recorded upon excitation at different wavelengths, subject to change when adding heavy metal ions to the solution. It was found that the efficiency of fluorescence quenching differed both for different probes and for excitation wavelengths corresponding to S_0_–S_2_ and S_0_–S_1_ transfers of electrons in the dye’s energy states. The observed differences in the changes of the spectra allowed us to generate a multisensor vector signal for selective recognition of various heavy metal ions employing the LDA algorithm.

This approach has promise because it allows one to use characteristics of complex photophysical processes occurring in the dyes composing the array as features to advance the selectivity of the ion detection. Still, further work is required to estimate the performance of the probe array under various interferences, at a long-term scale, according to specific practice applications. The mixes of various ions are also of special interest. With this purpose, other fluorescence probes should also be considered in the composition of the array.

## Data Availability

All relevant data are contained within the article; further data are available from the corresponding author under request.
